# Whole-genome identification and functional analysis of grape bHLH transcription factors: new insights into the regulation of monoterpene biosynthesis

**DOI:** 10.3389/fpls.2025.1653650

**Published:** 2025-09-16

**Authors:** Qian Sun, Zhenhua Liu, Xiaoyue Wang, Ailing Yan, Jiancheng Ren, Huiling Wang, Lei Sun

**Affiliations:** ^1^ Institute of Forestry and Pomology, Beijing Academy of Agriculture and Forestry Sciences, Beijing, China; ^2^ Beiing Key Laboratory of Forestry Food Processing and Safety, Department of Food Science, Beijing Forestry University, Beijing, China; ^3^ Key Laboratory of Biology and Genetic Improvement of Horticultural Crops (North China), Ministry of Agriculture and Rural Affairs, Beijing, China

**Keywords:** grape, bHLH gene family, bioinformatics, gene expression, monoterpene accumulation, subcellular localization, transient overexpression

## Abstract

Basic/helix-loop-helix (bHLH) transcription factors play critical roles in the regulation of plant secondary metabolism, but the molecular mechanisms involved in monoterpene synthesis by the grape bHLH family have not been systematically resolved. In this research, 12 *VvbHLHs* were detected in the grape genome, encoding 468–696 amino acids with molecular masses ranging from 52.5 to 77.5 kDa. Phylogenetic analyses showed that grapevine bHLH family members are closely related to dicotyledonous plants such as *Pyrus bretschneideri* and *Malus domestica*. These *VvbHLHs* were mainly localized in the nucleus, and their promoter regions were enriched for cis-acting elements linked to developmental stages and stress resistance. Spatio-temporal expression profiling indicated that *VvbHLHs* were significantly upregulated during fruit ripening, with the expression of *VvbHLH9* (*VvbHLH041*) significantly and positively correlated with monoterpene accumulation. Subcellular localization analysis showed that VvbHLH9 was localized in the nucleus; and transient overexpression assays revealed that *VvbHLH9* significantly activated monoterpene skeleton genes (*VvDXS1* and *VvDXS4*) and synthase genes (*VvTPS31*, *VvTPS32*, and *VvTPS35*) to promote the accumulation of monoterpene metabolites, such as terpinolene, allo-ocimene, nerol, geraniol, cis-furan linalool oxide and linalool. Notably, *VvbHLH9* lacked the typical bHLH-MYC_N structural domain, suggesting that it may affect the monoterpene metabolic network through a non-classical regulatory mechanism. The present study uncovers the modulatory function of grapevine bHLHs on the synthesis of monoterpene metabolites, providing new targets for grapevine fruit quality improvement and molecular breeding.

## 1 Introduction

As an important fruit crop widely cultivated around the world, grapevine (*Vitis vinifera* L.) is not only used for fresh consumption, winemaking and food processing, but also for pharmaceutical and industrial applications due to its richness in polyphenols, terpenoids and other active components (Chedea et al., 2022; Sabo et al., 2022). Recent studies have shown that the market value of grapes is strongly tied to the presence of metabolites (Wang et al., 2022a; Fan et al., 2023). Terpenoids, among the most abundant plant secondary metabolites, not only impart unique sensory characteristics to grapes but also have gained recognition for their remarkable therapeutic properties, include antimicrobial and anticancer properties (Mele et al., 2021). In addition, terpenoids are key regulators of plant developmental stages and stress resistance (Boncan et al., 2020; Wang et al., 2022b). However, terpenoids generally accumulate at low levels in plants, and how to optimize their synthetic pathways through genetic engineering has become an vital research direction to enhance the economic traits of grapes and develop terpenoid resources.

Transcription factors can achieve precise regulation of biosynthetic pathways through specific interactions with cis-acting elements in the promoters of target genes (Liu et al., 2022a). bHLH is the second largest gene family after MYB, and its members are crucial for light signal transduction, response to adversity, and secondary metabolism regulation through the formation of homo- or heterodimers (Ke et al., 2020b). Typical bHLH proteins contain two functional domains: the N-terminal DNA-binding domain consisting of 10–20 basic amino acids, which recognizes the E-box (CANNTG) sequences in the promoters of target genes; and the C-terminal HLH domain, which mediates protein dimerization through hydrophobic interactions, thereby regulating downstream gene transcription (Zhang et al., 2020). Up to now, the phylogenetic history of the bHLHs in the plant kingdom has been extensively investigated, including the model plant *Arabidopsis* (Toledo-Ortiz et al., 2003) and tobacco (Bano et al., 2021), as well as the common crops maize (Zhang et al., 2018b), barley (Ke et al., 2020a), foxtail millet (Fan et al., 2021), populus trichocarpa (Zhang et al., 2024a), and grape (Gao et al., 2019). For example, at least 162 bHLHs have been previously identified in *Arabidopsis*, which can be categorized into 12 different subfamilies, bHLH I to XII (Heim et al., 2003). In addition, another study identified 159 bHLH protein-coding genes in the tomato genome, and the *SlbHLH* genes were classified into 21 subfamilies by phylogenetic tree analysis (Sun et al., 2015). These plant bHLHs orchestrate development and adaptation to environmental stresses.

Numerous investigations have elucidated the functions of bHLHs across diverse plant species, demonstrating that certain bHLHs are key regulators of secondary metabolic pathways, such as anthocyanin, terpenoid, and alkaloid biosynthesis (Hichri et al., 2011). As a major class of transcriptional regulators, bHLHs participate in modulating terpene biosynthesis in plant systems, but most studies have been limited by the fact that they have only been carried out in model plants such as *Arabidopsis* (Hao et al., 2021). For many years, bHLHs have been found in *Arabidopsis*. For instance, the *Arabidopsis* bHLH transcription factor *AtMYC2* binds directly to the promoters of *AtTPS21* and *AtTPS11*, activating their expression and promoting the synthesis of sesquiterpenes (Hong et al., 2012). In addition, a regulatory role of *bHLHs* has been found in the terpene MVA pathway in plants. Mertens et al. (2016) identified two JA-induced *bHLHs TSAR1* and *TSAR2* in Medicago truncatula, which can activate the relevant genes of the MVA pathway through different activation modes, resulting in enhanced accumulation of triterpene saponins. In grapevine, scarce studies have predominantly emphasized the participation of bHLHs in metabolic control and growth processes. A recent investigation identified *VdbHLH037* as a member of subfamily III (f), which interacts with anthocyanin biosynthesis-related genes and contributes to anthocyanin production in spine grapes (Li et al., 2021). This result indicates that some grape *bHLHs* are associated with the production of anthocyanins. However, studies investigating the role of bHLHs in grape monoterpene metabolism are still scarce.

This study conducted whole-genome analysis of the grape bHLHs, mapped the distribution of *bHLHs* on chromosomes and performed covariance analysis; made statistical predictions of the physicochemical properties of the proteins and constructed their three-dimensional structural models. Phylogenetic evolutionary trees were constructed by sequence comparison of bHLH protein family members from seven species; and separate developmental evolutionary trees, gene structures and motif analyses were performed for *VvbHLHs* from grapevine. In addition, the high monoterpene variety ‘Ruidu Xiangyu’ at different developmental periods was selected as the test material for the study, and the expression pattern of grape *VvbHLHs* and its relationship with monoterpene synthesis were further explored by RT-qPCR analysis and GC-MS analysis. Especially importantly, we discovered for the first time that the expression level of *VvbHLH9* displayed a pronounced positive linkage with monoterpene production, and preliminarily explored its function through subcellular localization analysis and transient overexpression assays. The current results offer fresh perspectives into the regulatory mechanisms of the grape bHLHs, and offer theoretical support for enhancing grape quality through transcription factor engineering.

## 2 Materials and methods

### 2.1 Plant materials

The material was ‘Ruidu Xiangyu’ grape variety, taken from Beijing Forestry and Fruit Tree Research Institute of Forestry and Pomology, Beijing Academy of Agriculture and Forestry Sciences. Row spacing 2.5 m, plant spacing 0.75 m open field cultivation, inclined horizontal dragon vine, single-arm hedge shaping. Conventional cultivation and management measures were used. 25 May 2024, ‘Ruidu Xiangyu’ grapes bloomed, and fruit samples were collected 30 d after flowering (25 June). Samples were then taken at 15 d intervals until 10 August 2024 (ripening), a total of four times, labelled S1 (young fruit), S2 (expansion), S3 (veraison) and S4 (ripening), respectively. Sampling should be done on both the shaded and sunny sides, and on the shoulders, center and top of each cluster.

### 2.2 Identification and screening of grape *bHLH*

Firstly, we downloaded the protein data of grape in Emsembl plants (ftp://ftp.ensemblgenomes.org/) and obtained the protein sequence of *Arabidopsis* bHLH transcription factor in PlantTFDB5.0. The analysis was conducted using a combination of the HMMER 3.0 software and the native Blastp method, with a limited E-value of 1 × e^-10^ for HMMER 3.0 and an E-value set at 1 × e^-5^ for Blastp. Subsequently, BatchCD-search in the NCBI online website (https://www.ncbi.nlm.nih.gov/) was used to retrieve the conserved structural domains of the bHLH family and collected all the results of the query with E-value less than 0.01, which were the predicted grape *VvbHLH* genes. The conserved structural domains of *VvbHLHs* were analyzed using the sequence analysis software DNAMAN 6.0 and visualized using Tbtools (v1.098774) (Chen et al., 2020).

### 2.3 Chromosomal localization and covariance analysis of grape *bHLH*

With the help of (gff3) file of grape genome annotation information, the bHLHs were located in the chromosome, and then the Gene location visualize from gff3 function of Tbtools (v1.098774) was used to visualize and analyze the structural results of the *bHLH* gene. Gene replication events were analyzed by the MCScanX program and KaKs_Calculator2.0 (Wang et al., 2024), and grape bHLH family members as well as covariance between *VvbHLHs* and *Arabidopsis AtbHLHs* were visualized using Tbtools (v1.098774).

### 2.4 Physicochemical property analysis and spatial structure prediction of grape bHLH

Using ProtParam software (https://web.expasy.org/protparam) analyze the physicochemical properties of bHLHs in grapes, including the number of amino acids, theoretical isoelectric point, and molecular weight (Eslami Bojnourdi et al., 2023). The subcellular localization of grape bHLHs was predicted using the WoLFPSORT (https://wolfpsort.hgc.jp/) (Salih et al., 2021). Prediction of protein secondary structure of grape bHLHs using SOPMA tool (http://npsa-pbil.ibcp.fr/); the tertiary structure of grape VvbHLH protein was predicted and analyzed using the SWISS-MODEL (https://swissmodel.expasy.org/) website (Liang et al., 2023).

### 2.5 Phylogenetic analysis, gene structure and conserved motif analysis

The bHLH sequences of grape and the bHLH sequences of *Arabidopsis*, *Zea mays*, *Pyrus bretschneideri*, *Malus domestica*, *Populus trichocarpa* and *Oryza sativa* species were subjected to multiple sequence comparisons using the MUSCLE tool in the MEGA11 software, and the phylogenetic tree was drawn by the neighbor-joining method (Zhang et al., 2021), and the constructed phylogenetic tree was trimmed and beautified using the Evolview (http://www.evolgenius.info/evolview/#/) online tool to modify and beautify the constructed phylogenetic tree. Gene sequence intron and UTR analyses were performed on the GSDS website (http://gsds.gao-lab.org/). Protein motif analysis was performed on the MEME website (http://meme-suite.org/tools/meme) (Zhang et al., 2021). The phylogenetic tree, conserved structural domains and gene structure results of *VvbHLH* were visualized in evolutionary tree order using Tbtools (v1.098774).

### 2.6 Cis-element analysis of grape bHLH promoter

The 2000 bp upstream of the start codon of the bHLH family gene was extracted from the grape genome file as the promoter region, and promoter cis-element analysis was performed using PlantCARE (http://bioinformatics.psb.ugent.be/webtools/plantcare/html) (Zhang et al., 2024c). Tbtools (v1.098774) was used for promoter visualization of the results.

### 2.7 Determination of monoterpene content

Free-state monoterpenes were extracted and determined with reference to the method of Liu et al. (2022b). Retention indices (RIs) and mass spectral information were obtained by calculation using the Automated Mass Spectrometry Deconvolution and Identification Software (AMDIS), and the results of mass spectral analyses were searched by matching the results with the NIST Substance Retrieval Spectral Library, as well as the retention indices of the substance standards to identify the substances. The concentration determination of grape terpenoids is carried out through a standard curve. The standard curves were prepared and plotted with reference to the method of Zhou et al. (2022). For monoterpenes for which no standard curve is available, quantitative analyses should be carried out by means of standard curves for substances with a similar number of carbon atoms and similar chemical structure (**Supplementary Table 1**).

### 2.8 RNA extraction and RT-qPCR analysis

Primers specific for the grape bHLH family gene were designed using Premier 6 software (**Supplementary Table 2**), and total RNA was extracted using the TIANGEN RNAplant kit (TIANGEN, China). The yield and quality of RNA were determined using a NanoDrop spectrophotometer (ND-7000, NanoDrop Technologies, Inc., USA). The cDNA was synthesized using All-in-one 1st Strand cDNA Synthesis Super Mix (gDNA Purge) Reverse Transcription Kit (Novoprotein, China). cDNA was diluted 5-fold at a concentration of 200 ng/uL and used for RT-qPCR reaction. RT-qPCR Reactions were performed using SYBR qPCR mixture (GenStar, China) and CFX-connected real-time PCR detection system (Bio-Rad, USA). The PCR cycles were: 95°C for 2 min, 40 cycle numbers, 95°C for 15 s, and 60°C for 34 s. Each reaction was performed with 3 biological replicates. VvUbiquitin was used as an internal reference gene. Relative expression levels were counted using the 2^-ΔΔCT^ method (Harshitha and Arunraj, 2021).

### 2.9 Correlation analysis between grape *bHLH* gene expression level and monoterpene content

Based on the changes of monoterpene content in grape berries and the relative expression of grape bHLHs at different periods, Pearson correlation analysis was performed using SPSS 27.0 (SPSS, USA). The significance of correlations was assessed at P < 0.05 and P < 0.01 levels, with adjustments for multiple testing using the Benjamini-Hochberg false discovery rate (FDR) method. Heatmaps were drawn using ChiPlot (https://www.chiplot.online/) to visualize the correlation between the changes of monoterpene content and the gene expression results were visualized.

### 2.10 *VvbHLH9* gene cloning, plasmid construction and subcellular localization

We generated cDNA by reverse transcription of total RNA from grape berries and cloned the *VvbHLH9* gene. Subcellular localization of the *VvbHLH9* gene was performed in order to determine the true positional information of the gene. We constructed a recombinant plasmid incorporating the GFP tag and the VvbHLH9 protein, and injected the recombinant vector into *N. benthamiana* leaves using Agrobacterium-mediated transformation, and used the empty PCAMBIA2300-GFP vector with a GFP tag as a control. After overnight dark incubation, the culture was then cultivation under light for 12 hours. About 3 days after leaf infiltration, the leaves were placed on slides and GFP fluorescence in transgenic *N. benthamiana* leaf cells was observed using a FV10-ASW laser confocal microscope. GFP stands for green fluorescence field, CHI stands for chloroplast autofluorescence field, DAPI stands for DAPI field (cytosolic staining), DIC stands for bright field, and Merge stands for superimposed field.

### 2.11 Transient overexpression in leaves

The fragment of *VvbHLH9* was cloned into pCAMBIA2300-GFP vector, and the recombinant vector was used for transient overexpression analysis. Fresh ‘Alden’ variety grapevine leaves of similar size and without obvious mechanical damage were selected for infestation, and six leaves were infested with each sample as six technical parallels, and the same number of leaves were infested with Agrobacterium transfected into the pCAMBIA2300-GFP empty vector as the control group. The bacteriophage solution was incubated for 12 h until the OD_600_ was 0.6-1.0. The bacteriophage OD_600_ was adjusted to 0.4 using infestation buffer [MES 2.132 g/L, MgCl_2_·6H_2_O 2.033 g/L, sucrose 5 g/L, and acetosyringone at a final concentration of 200 mg/L, pH 5.9], and left to stand for 4 h. After infiltration, the leaves were incubated under low temperature and light protection for 3 days. The expression levels of *VvbHLH9* and monoterpene synthesis-related genes were obtained by RT-qPCR, and the monoterpene substances were detected by GC-MS. The primers are shown in **Supplementary Table 3**.

## 3 Results

### 3.1 Identification of grape *bHLH* gene

**Figure 1:**
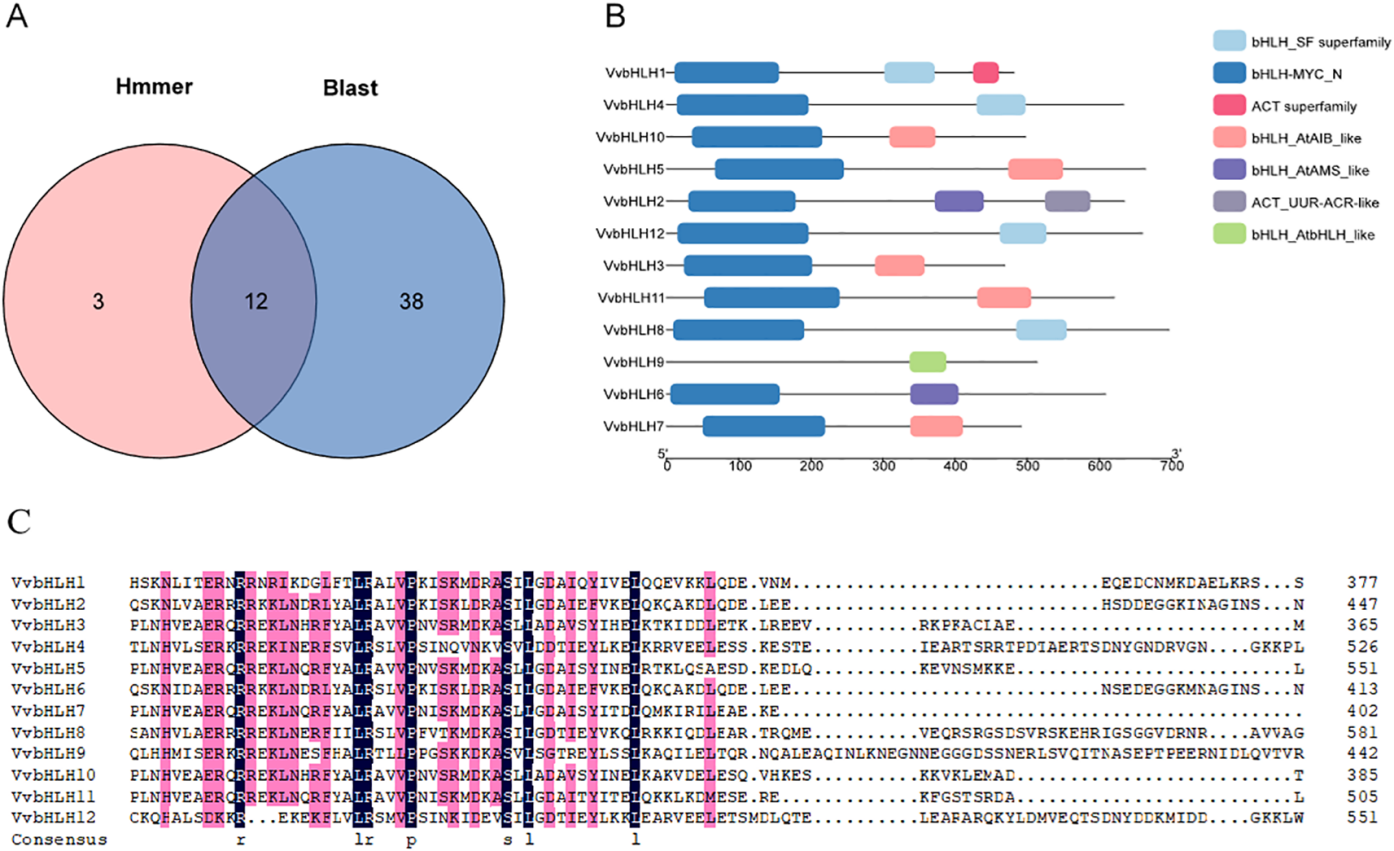
Identification of grape VvbHLHs and analysis of protein conserved structural domains. **(A)** Identification results of grape VvbHLH gene family. Hmmer: genes identified by HMMER software; Blast: genes compared by Tbtools software. **(B)** Analysis of grape VvbHLH protein conserved structural domains. **(C)** Comparative analysis of HLH conserved structural domains of grape VvbHLH gene family members. Black and pink colours indicate 100% and 75% amino acid conservation, respectively; hyphens indicate intervals.

Using HMMER software and TBtools local BLAST analysis, we identified 12 grape bHLH protein sequences that met the screening criteria (**Figure 1A**). According to the chromosomal location, these bHLH sequences were named VvbHLH1~VvbHLH12. Based on the conserved structural domains, we classified the grape bHLH family into bHLH_SF superfamily, bHLH-MYC_N, ACT superfamily, bHLH_AtAIB_like, bHLH_ AtAMS_like, ACT_UUR-ACR-like, and bHLH_AtbHLH_like, which are seven types. Except for VvbHLH9, the remaining 11 VvbHLHs contain the bHLH-MYC_N structural domain at the N-terminus (**Figure 1B**). Analysis of the HLH conserved structural domains of the 12 grape bHLHs using DNAMAN software showed that the C-terminal HLH structural domains were enriched in the hydrophobic amino acids leucine (L) and proline (P), of which, L was highly conserved and was important for maintaining the stability of the secondary structure of the bHLH protein (**Figure 1C**).

### 3.2 Chromosomal localization and covariance analysis of grapevine *bHLH* gene

**Figure 2:**
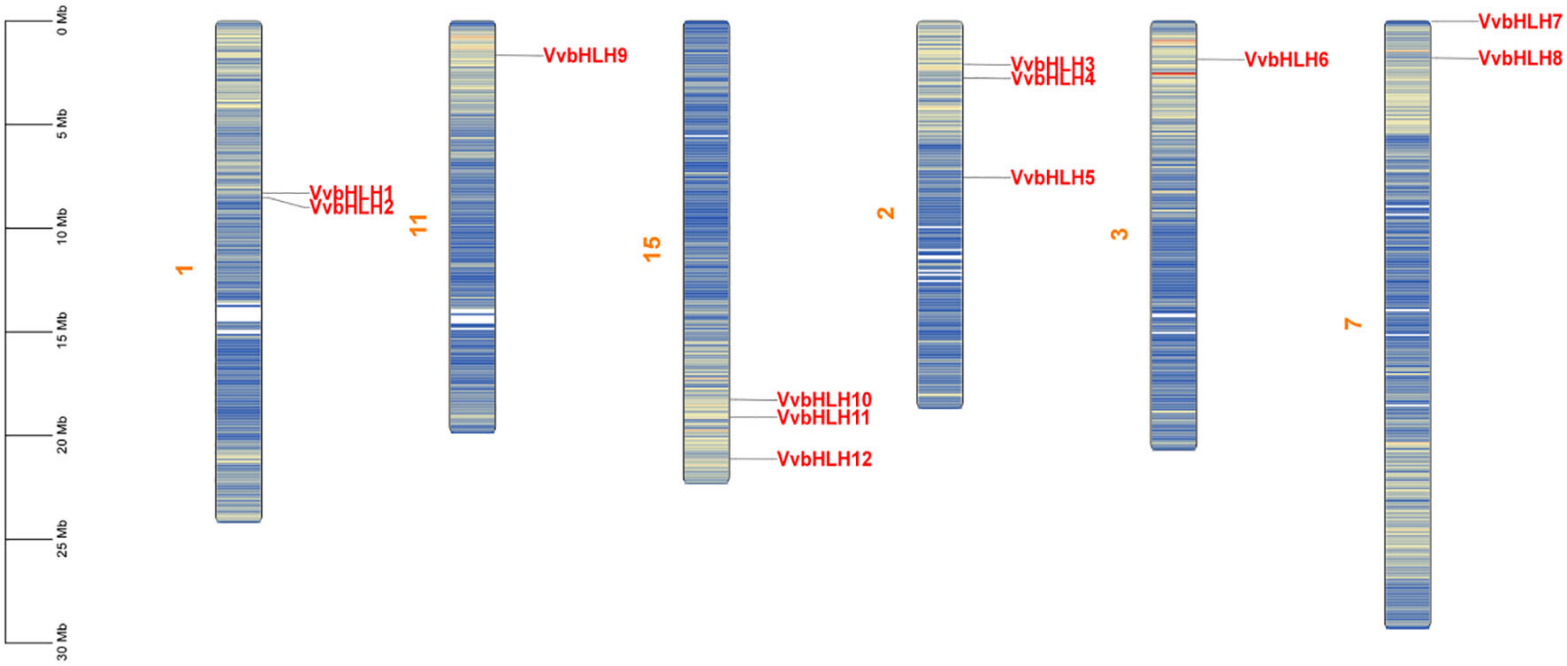
Chromosome distribution pattern of grape bHLH gene family members. Yellow characters are chromosome names, red characters are gene names, the chromosome colour gradient from blue to red represents gene density from low to high, and blank regions indicate the lack of gene distribution on the chromosome.

To further confirm the evolutionary duplication events of *VvbHLH*, we analyzed their chromosomal distribution and syntenic relationships. According to the grape genome annotation gff3 file, the 12 grape *VvbHLH*s identified were localized to six grape chromosomes (**Figure 2**). The 12 *VvbHLHs* were irregularly distributed, and the genes were distributed in different densities on different chromosome skeletons. Among them, chromosomes 2 and 15 in grapes contained the highest number of *bHLHs* with three; chromosomes 1 and 7 both contained two *bHLH* genes; and chromosomes 3 and 11 contained the lowest number of *bHLH* genes with one.

**Figure 3:**
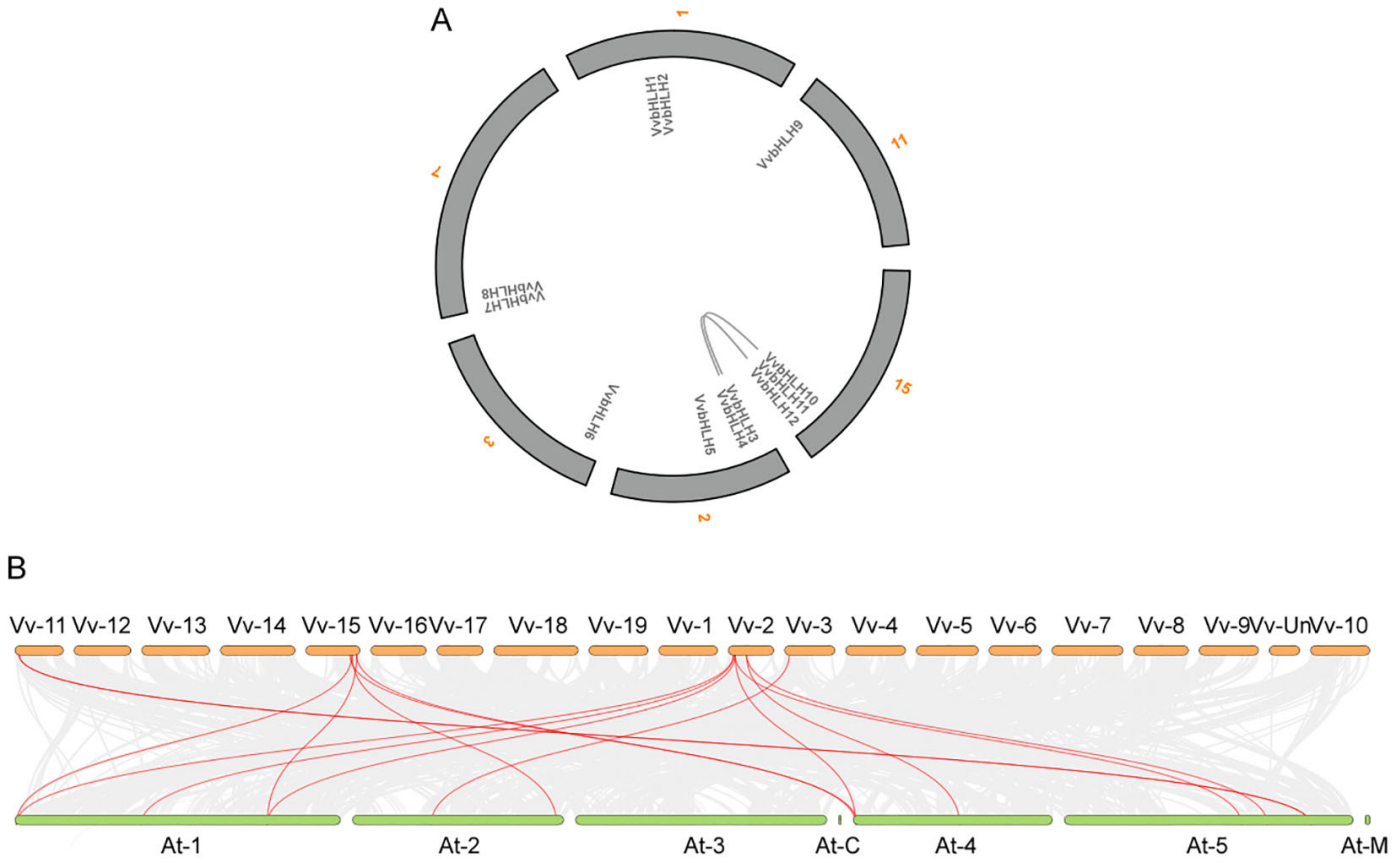
Analysis of covariance of *VvbHLH* within grape and between grape and *Arabidopsis*. **(A)** Covariance analysis of grape *VvbHLH*. The gray text in the figure represents 12 *bHLH* genes, and the orange numbers represent different chromosomes. **(B)** Covariance analysis of *VvbHLH* between grape and *Arabidopsis*. Vv represents *Vitis vinifera*, At represents *Arabidopsis*, and the numbers represent different chromosomes.

The collinearity analysis revealed that the two pairs of grape *bHLHs* exhibit a syntenic relationship (**Figure 3A**; **Supplementary Table 4**), *VvbHLH3* and *VvbHLH10*, and *VvbHLH4* and *VvbHLH12*, which can be inferred to have been formed through the replication of large segments of the genes. To further understand the evolutionary links among the *bHLHs*, we conducted collinearity analysis of *bHLHs* between grapes and *Arabidopsis* (**Figure 3B**; **Supplementary Table 4**). It was found that a total of eight *VvbHLH* genes in grape shared covariance with *Arabidopsis bHLH* genes. Among them, three *VvbHLH* genes showed homology with one *Arabidopsis bHLH* gene, including *VvbHLH6*, *VvbHLH9*, and *VvbHLH10*; four *VvbHLH* genes showed homology with two *Arabidopsis bHLH* genes, including *VvbHLH3*, *VvbHLH5*, *VvbHLH11*, and *VvbHLH12*; one *VvbHLH* gene showed homology with three *Arabidopsis bHLH* genes, *VvbHLH4* (**Supplementary Table 4**).

### 3.3 Physicochemical property analysis of grape bHLH family proteins

Analysis of the physicochemical properties of grape bHLH proteins showed (**Table 1**) that the length of grape bHLHs ranged from 468 aa (VvbHLH3) to 696 aa (VvbHLH8). The molecular mass of the 12 bHLHs ranged from 52,465.4 Da to 77,531.46 Da, with the highest molecular mass of the VvbHLH8 protein, and the lowest molecular mass of the VvbHLH3 protein. VvbHLH8 protein had the highest molecular mass and VvbHLH3 protein had the lowest. The pI of the grape bHLHs ranged from 5.01 (VvbHLH2) to 7.93 (VvbHLH1), and 11 bHLH proteins had pIs between 5 and 7, indicating that most of the grape bHLH family proteins had theoretically acidic pIs. The instability coefficients ranged from 38.2 (VvbHLH7) to 66.03 (VvbHLH8), indicating that most of the members were unstable proteins (II > 40); only two belonged to stable proteins (II < 40), with instability coefficients of 38.2 (VvbHLH7) and 39.48 (VvbHLH10), respectively. The fat coefficients were 67.51 (VvbHLH5) to 88.52 (VvbHLH1). In addition, the GRAVY index of all bHLH proteins was less than 0, revealing that these proteins exhibit predominantly hydrophilic properties, but the hydrophilicity varied among different proteins. Subcellular localization prediction revealed that most of the bHLHs were subcellularly localized in the nucleus and a few in the chloroplasts.

**Table 1:** Physicochemical properties of grape bHLH protein.

Gene name	Gene ID	Gene location	Number of amino acid (aa)	Molecular Weight (Da)	Theoretical pI	Instability index	Aliphatic index	GRAVY index	Subcellular localization
VvbHLH1	Vitvi01g04182	Chr1:8297887-8300246	481	54375.31	7.93	53.71	88.52	-0.445	Chloroplast
VvbHLH2	Vitvi01g00746	Chr1:8526024-8528767	634	71004.64	5.01	47.67	78.11	-0.506	Nucleus
VvbHLH3	Vitvi02g00231	Chr2:2093930-2095605	468	52465.4	6.15	42.33	81.86	-0.427	Nucleus
VvbHLH4	Vitvi02g00317	Chr2:2751661-2756552	633	71124.2	5.12	52.52	86.52	-0.48	Nucleus
VvbHLH5	Vitvi02g00698	Chr2:7555494-7557485	663	72774.89	5.52	53.1	67.51	-0.599	Nucleus
VvbHLH6	Vitvi03g00157	Chr3:1855132-1857690	608	67745.57	5.1	51.42	74.59	-0.589	Chloroplast
VvbHLH7	Vitvi07g00002	Chr7:27048-29059	491	54750.34	5.97	38.2	83.91	-0.369	Nucleus
VvbHLH8	Vitvi07g00139	Chr7:1785693-1791218	696	77531.46	5.63	66.03	69.66	-0.671	Nucleus
VvbHLH9	Vitvi11g00165	Chr11:1657895-1660637	513	57815.32	6.69	65.82	73.63	-0.505	Nucleus
VvbHLH10	Vitvi15g00906	Chr15:18261961-18263454	497	54587.91	5.77	39.48	82.82	-0.374	Nucleus
VvbHLH11	Vitvi15g00948	Chr15:19114417-19116915	620	69218.92	6	45.59	72.82	-0.525	Nucleus
VvbHLH12	Vitvi15g01124	Chr15:21113482-21119462	659	74351	5.1	51.63	80.33	-0.517	Nucleus

### 3.4 Spatial structure analysis of grape bHLH protein

**Figure 4:**
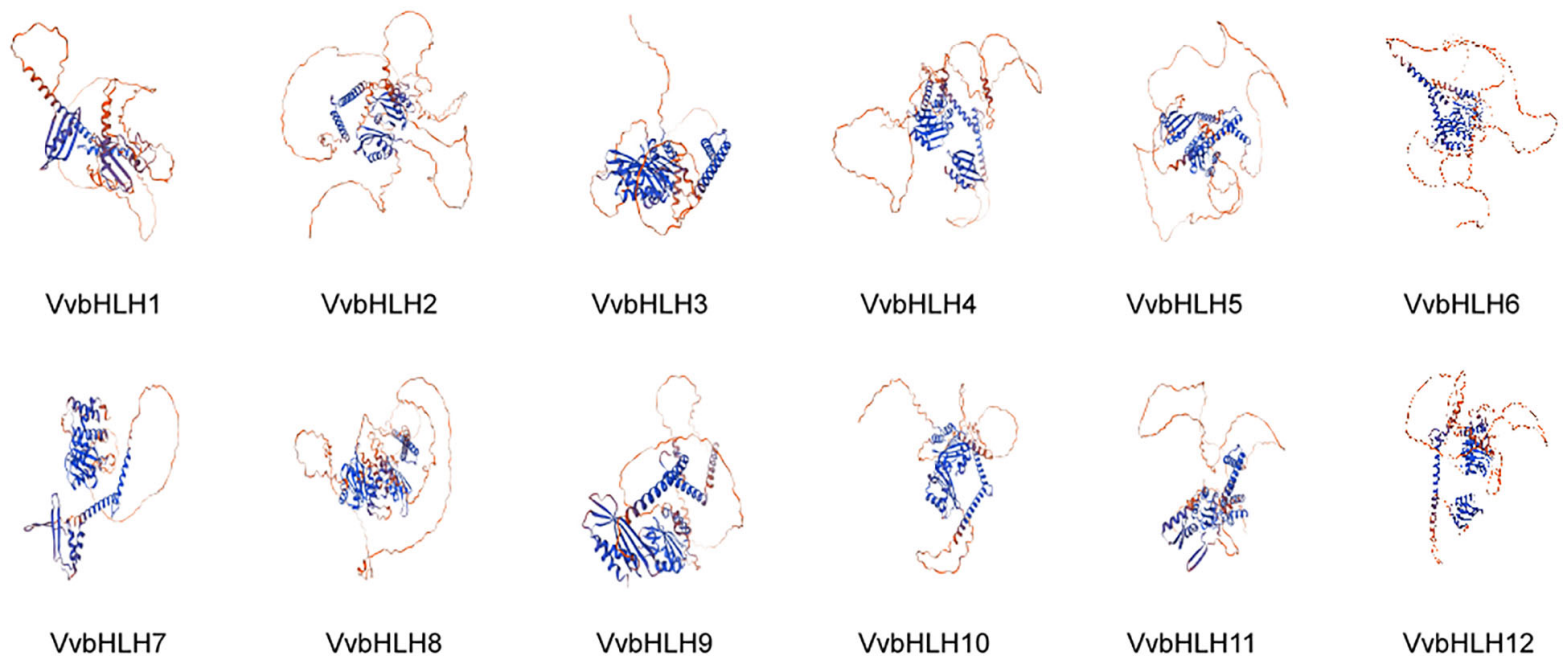
Predicted tertiary structure of grape bHLH protein.

The amino acid sequences of the bHLHs were predicted and analyzed for secondary structure by SOPMA. Research has found that the secondary structure of grape bHLHs mainly consisted of α-helix (Hh), random coil (Cc), β-turn (Tt) and extended strand (Ee) (**Table 2**). Among them, random coils and α-helices accounted for the largest proportion, and β-turns accounted for the smallest proportion. α-helices structures had a number proportion of 30.59% (VvbHLH6) to 39.18% (VvbHLH9), extended strand structures had a number proportion of 9.10% (VvbHLH12) to 13.44% (VvbHLH7), and β-turns structures had a number proportion of 1.51% (VvbHLH5) to 4.74% (VvbHLH4), and the proportion of the number of random coil structures is 45.22% (VvbHLH9) to 54.44% (VvbHLH6). The sequences of the grape bHLHs were analyzed for the prediction of tertiary structures through the SWISS-MODEL website. The analysis revealed that the model match similarity exceeded 30% for all members, and the protein structures of the same subfamily had high similarity, but there were also differences between individuals (**Figure 4**).

**Table 2:** Predicted secondary structure of grape bHLH gene family proteins.

Gene name	α-Helix (%)	Extended strand (%)	β-turn (%)	Random coil (%)
*VvbHLH*1	32.22%	10.19%	4.57%	53.01%
*VvbHLH*2	33.75%	10.25%	3.00%	53.00%
*VvbHLH*3	38.03%	11.54%	1.92%	48.50%
*VvbHLH*4	37.12%	10.58%	4.74%	47.55%
*VvbHLH*5	32.88%	12.67%	1.51%	52.94%
*VvbHLH*6	30.59%	10.86%	4.11%	54.44%
*VvbHLH*7	36.05%	13.44%	3.26%	47.25%
*VvbHLH*8	38.79%	9.63%	3.45%	48.13%
*VvbHLH*9	39.18%	12.87%	2.73%	45.22%
*VvbHLH*10	33.40%	12.47%	3.22%	50.91%
*VvbHLH*11	32.74%	13.39%	2.58%	51.29%
*VvbHLH*12	37.03%	9.10%	3.79%	50.08%

### 3.5 Phylogenetic analysis, gene structure and conserved motif analysis

**Figure 5:**
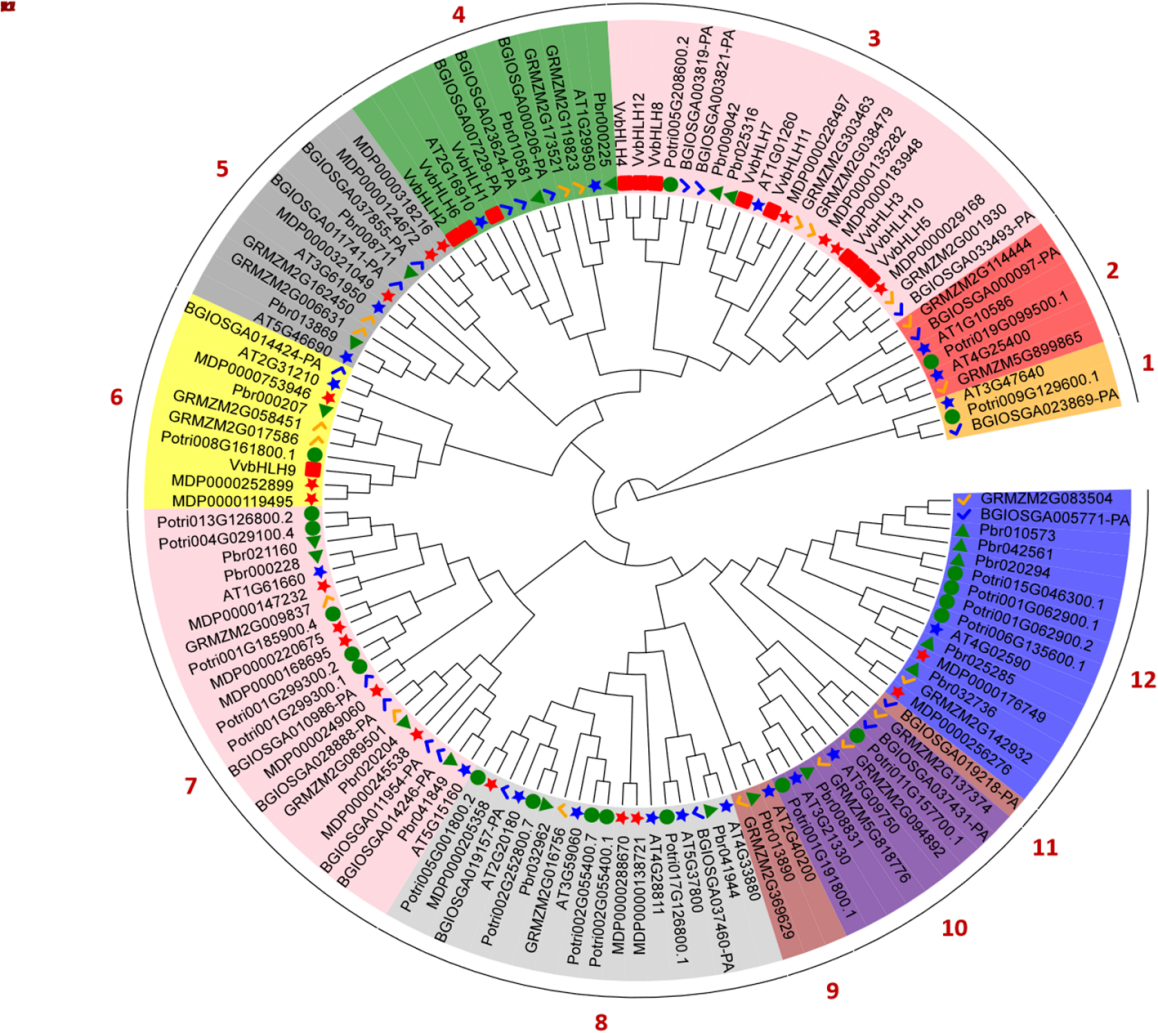
Phylogenetic evolutionary tree of bHLHs from Vitis vinifera, Arabidopsis, Zea mays, *Pyrus bretschneideri*, Malus domestica, Populus trichocarpa and Oryza sativa. Vv. Vitis vinifera; At. Arabidopsis thaliana; Zm. Zea mays; Pbr. *Pyrus bretschneideri*; Mdp. Malus domestica; Pot. Populus trichocarpa; Bgi. Oryza sativa.

To elucidate the phylogenetic relationship between grape and other plant bHLHs, we constructed an evolutionary tree by comparing the protein sequences of the grape bHLHs with those of *Arabidopsis*, *Zea mays*, *Pyrus bretschneideri*, *Malus domestica*, *Populus trichocarpa* and *Oryza sativa* (**Figure 5**). Based on evolutionary relationships, a total of 132 bHLHs from different species were classified into 12 subfamilies (Group 1 ~ Group 12), with the smallest subfamily, Group 11, containing only one member, and the largest subfamilies, Group 3 and Group 7, with 22 members. Phylogenetic analysis of grapevine bHLHs showed that the 12 VvbHLH protein sequences could be divided into 3 groups: Group 3 contained 8 VvbHLH proteins, Group 4 contained 3 VvbHLH proteins, and Group 6 contained 1 VvbHLH protein. As can be seen from the figure, all grape *VvbHLH* genes are always clustered together with those of *Pyrus bretschneideri*, *Malus domestica* and *Arabidopsis* with close affinity, while they are relatively distantly related to the monocotyledonous plants *Zea mays* and *Oryza sativa*.

**Figure 6:**
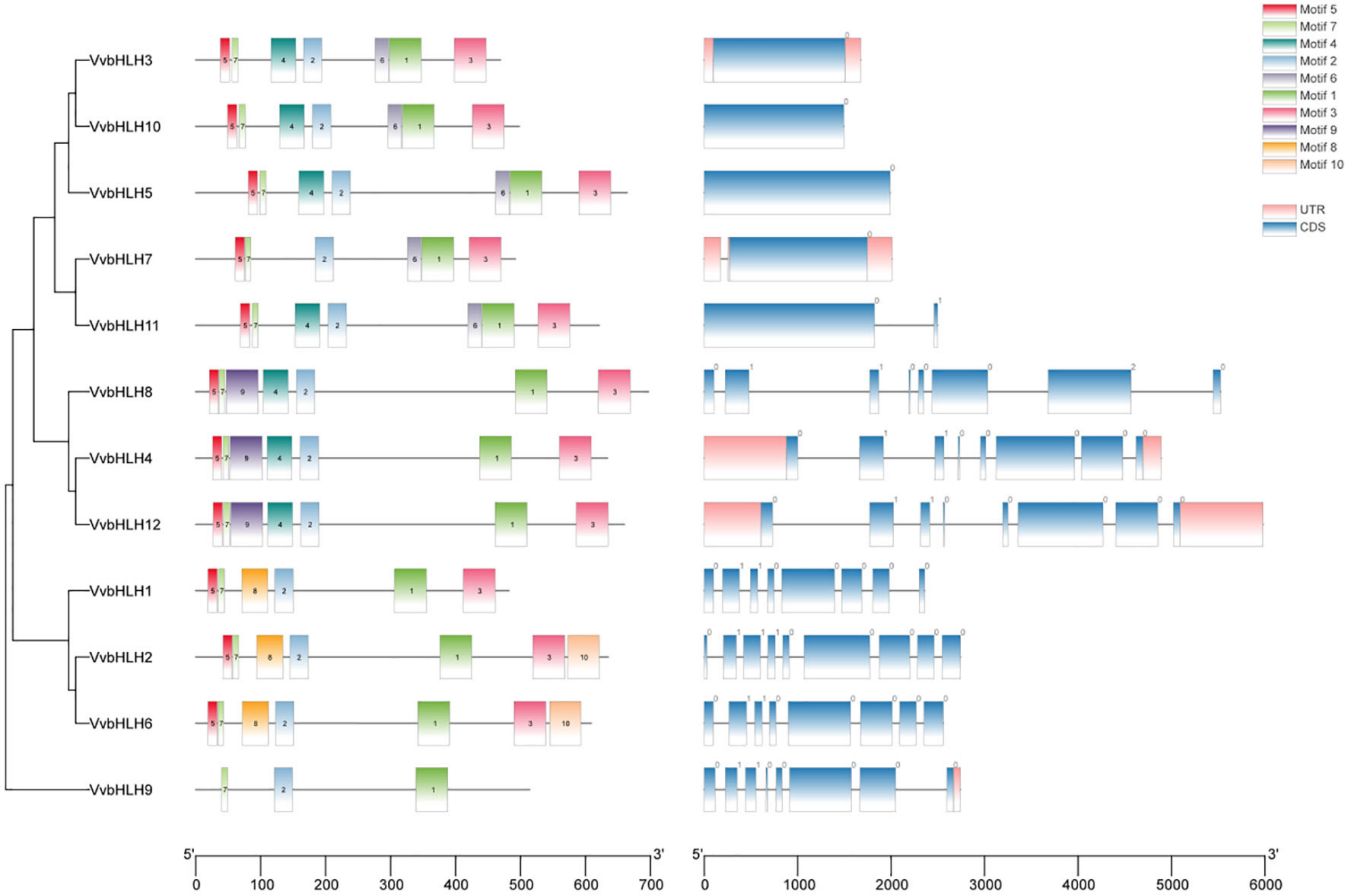
Phylogenetic analysis, gene structure and motif analysis of the grape bHLH protein family.

In order to better analyze the bHLHs of grapevine, the study was carried out with separate developmental evolutionary trees, gene structure and motif analyses (**Figure 6**). It can be seen that VvbHLHs include 3 classes, which aligning with the protein phylogenetic tree classification. It can be found that all grape bHLH family members have coding sequences (CDS), and all have untranslated regions (UTR) except *VvbHLH1*, *VvbHLH2*, *VvbHLH5*, *VvbHLH6*, *VvbHLH8*, *VvbHLH10* and *VvbHLH11*, and those that don’t may not be annotated. Meanwhile, grape bHLH family members all contain Motif 1, indicating the relative conservatism of the bHLH family. In addition, bHLH members containing the same kinds of conserved motifs are evolutionarily more closely related. Based on Motif differences, it was found that Group 3 subfamily members all contained Motif 1, Motif 2, Motif 3, Motif 5, and Motif 7; Group 4 subfamily members all contained Motif 1, Motif 2, Motif 3, Motif 5, Motif 7, and Motif 8; and Group 6 subfamily members all contained Motif1, Motif2 and Motif 7. It is hypothesized that the differences in motifs contained in the different branches may be one of the reasons for the functional divergence of VvbHLHs during the evolutionary process.

### 3.6 Cis promoter action elements of grape bHLHs

**Figure 7:**
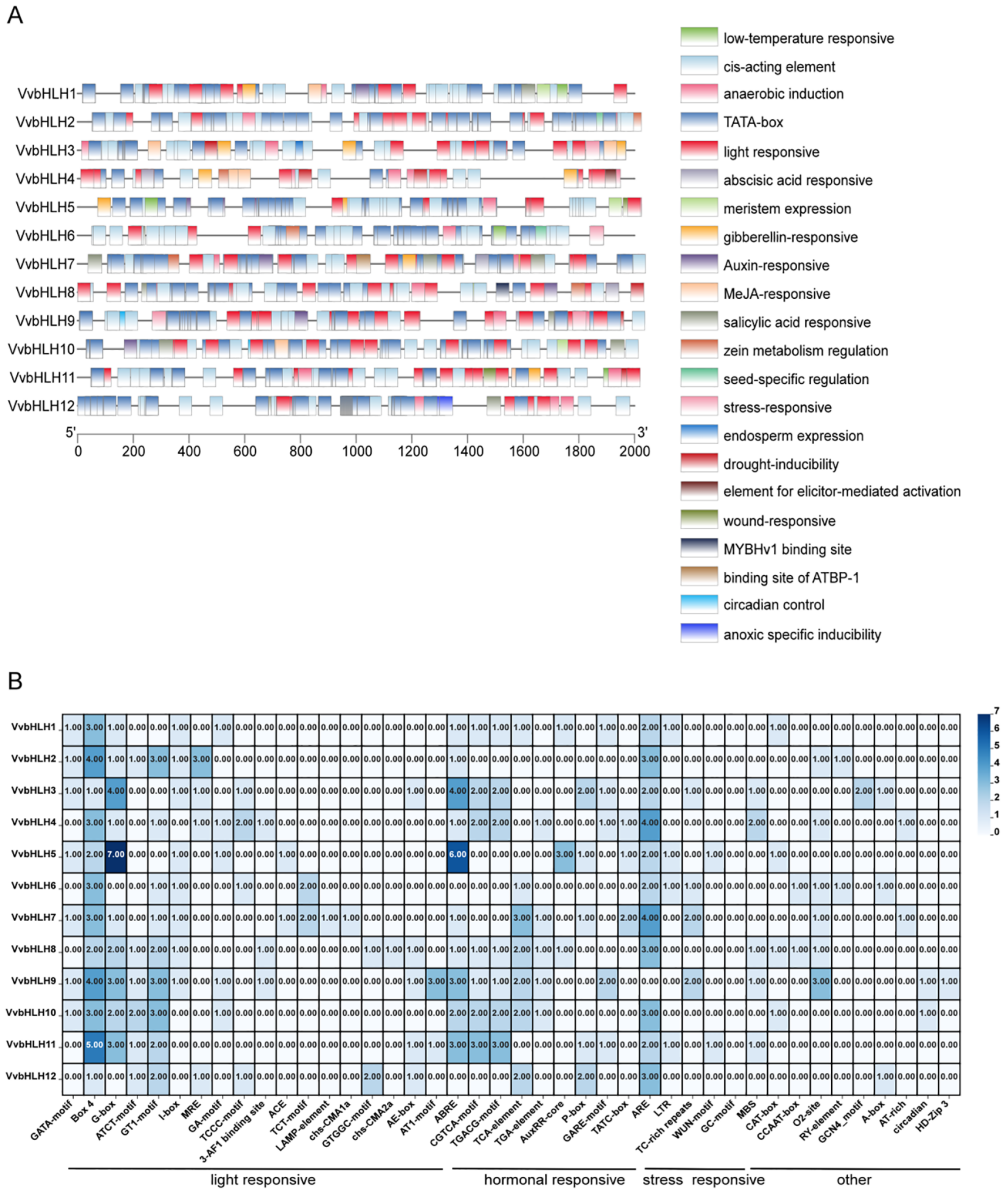
Analysis of the type and number of cis-acting elements in the promoter of grape bHLH gene. **(A)** Schematic diagram of cis-acting elements in the promoter of VvbHLH. **(B)** Heat map analysis of the number of cis-acting elements in the promoter of VvbHLH.

To understand the transcriptional regulation of grape *bHLHs*, in this study, we extracted the grape family gene sequences 2000 bp upstream of the start codon by using TBtools, and analyzed and counted the cis-acting elements in conjunction with the PlantCARE database, and then visualized and analyzed the results by using the TBtools after removing the redundant and irrelevant sequences manually. The results showed that the promoters of *VvbHLHs* differed in the type and number of elements contained in their promoters (**Figure 7**). The promoter region of the *VvbHLHs* contains many functional elements related to abiotic stresses, such as: circadian response elements, cis-regulatory elements involved in light response, cis-elements involved in low-temperature response, drought stress cis-responsive elements, and hypoxia-specific inducible elements (**Figure 7**). It was found that all bHLH family members contained light-responsive elements, and it was hypothesized that their functions were closely related to the light-responsive pathway. Hormone-related response elements accounted for more in grapes, with abscisic acid and methyl jasmonate response elements being the most numerous, with 23 and 24, respectively (**Figure 7A**). Therefore, it can be speculated that bHLHs are involved in the transmission of hormone signals such as auxin, abscisic acid, and methyl jasmonate. In addition, we found that six *VvbHLH* genes (*bHLH2*, *bHLH4*, *bHLH6*, *bHLH7*, *bHLH8*, and *bHLH9*) were involved in the regulation of zeinolysin metabolism in plants, which suggests that *VvbHLHs* are involved in metabolically regulated processes in plants (**Figure 7A**).

The study further analyzed the number of cis-acting elements in the VvbHLH promoter (**Figure 7B**). The results suggested that the major response elements included Box 4 and antioxidant response element (ARE), both of which were 1–5 and 2–4 in the VvbHLH promoter, respectively. Light-responsive elements mainly consisted of Box 4 and G-box elements, with VvbHLH2, VvbHLH9, and VvbHLH11 containing more than three Box 4 elements; VvbHLH5 contained up to seven G-box elements. The hormone response elements mainly consisted of abscisic acid response elements (ABRE), which were high in VvbHLH3, VvbHLH5, VvbHLH9, and VvbHLH11, with 4, 6, 3, and 3 elements, respectively. The stress response elements were mainly antioxidant response element (ARE) followed by defense and stress response element (TC-rich repeats) elements. Other acting elements mainly included maize alcohol soluble protein metabolic regulatory element (O2-site) and drought response element (MBS), etc.

### 3.7 Analysis of grapevine bHLHs expression patterns

**Figure 8:**
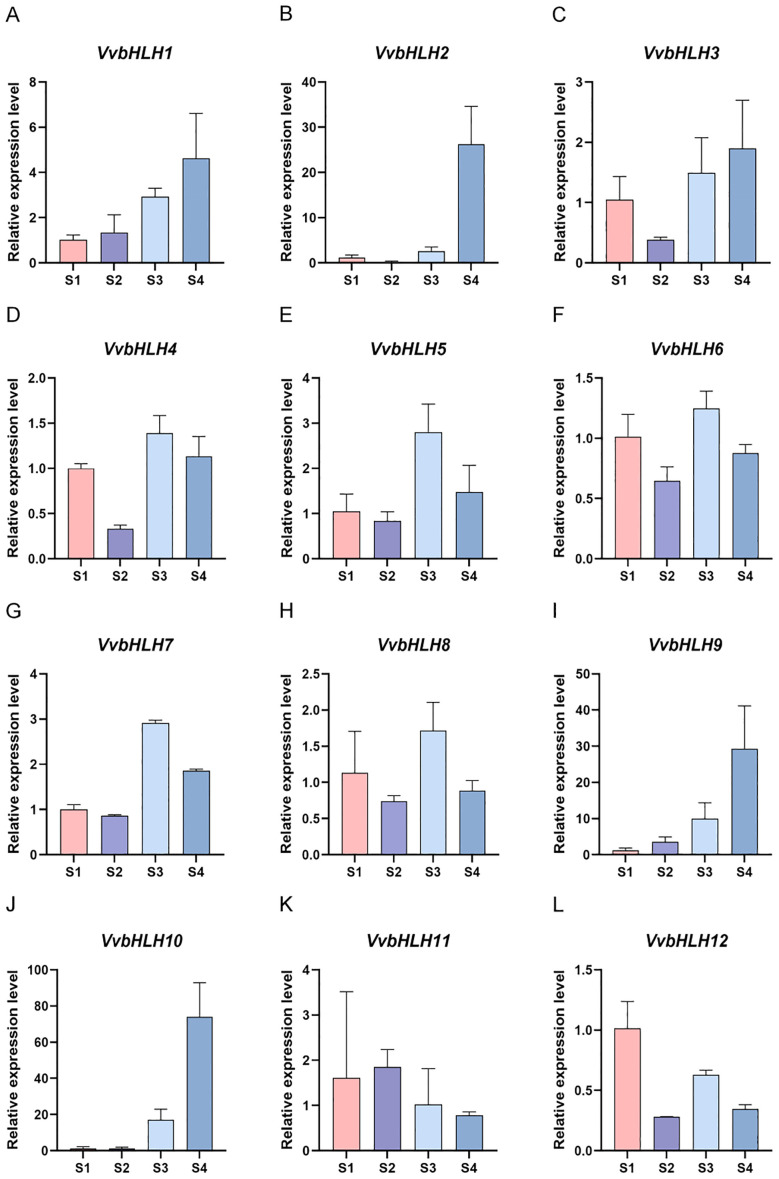
Expression pattern of bHLH gene family in ‘Ruidu Xiangyu’ grapes at different developmental stages. **(A–L)** Expression trends of 12 bHLH genes during four developmental stages (S1-S4).

We analyzed the spatio-temporal expression pattern of *VvbHLHs* during grapevine fruit development using RT-qPCR. Research has demonstrated that the expression of *VvbHLH2* had significant spatio-temporal dynamics compared to other VvbHLH genes during different developmental periods in the plant, and the gene showed a higher expression level during the S4 (ripening) period of the fruit (**Figure 8**). Among the 12 *VvbHLH* genes, five *VvbHLH* genes (*VvbHLH4*, *VvbHLH5*, *VvbHLH6*, *VvbHLH7*, and *VvbHLH8*) had a significantly high expression in the fruit during the S3 (veraison) period, when grapes were in the veraison stage, which was hypothesized to be possibly associated with the accumulation of monoterpenes in grapes (**Figure 8**). In addition, five *VvbHLH* genes (*VvbHLH1*, *VvbHLH2*, *VvbHLH3*, *VvbHLH9*, and *VvbHLH10*) were up-regulated in gene expression during the S4 period, the VvbHLH11 gene was up-regulated during the S2 (expansion) period, and the *VvbHLH12* gene was up-regulated during the S1 (young fruit) period. These findings reveal that these *VvbHLHs* exhibit distinct functions in development stages of grape berries.

### 3.8 Monoterpene content

**Figure 9:**
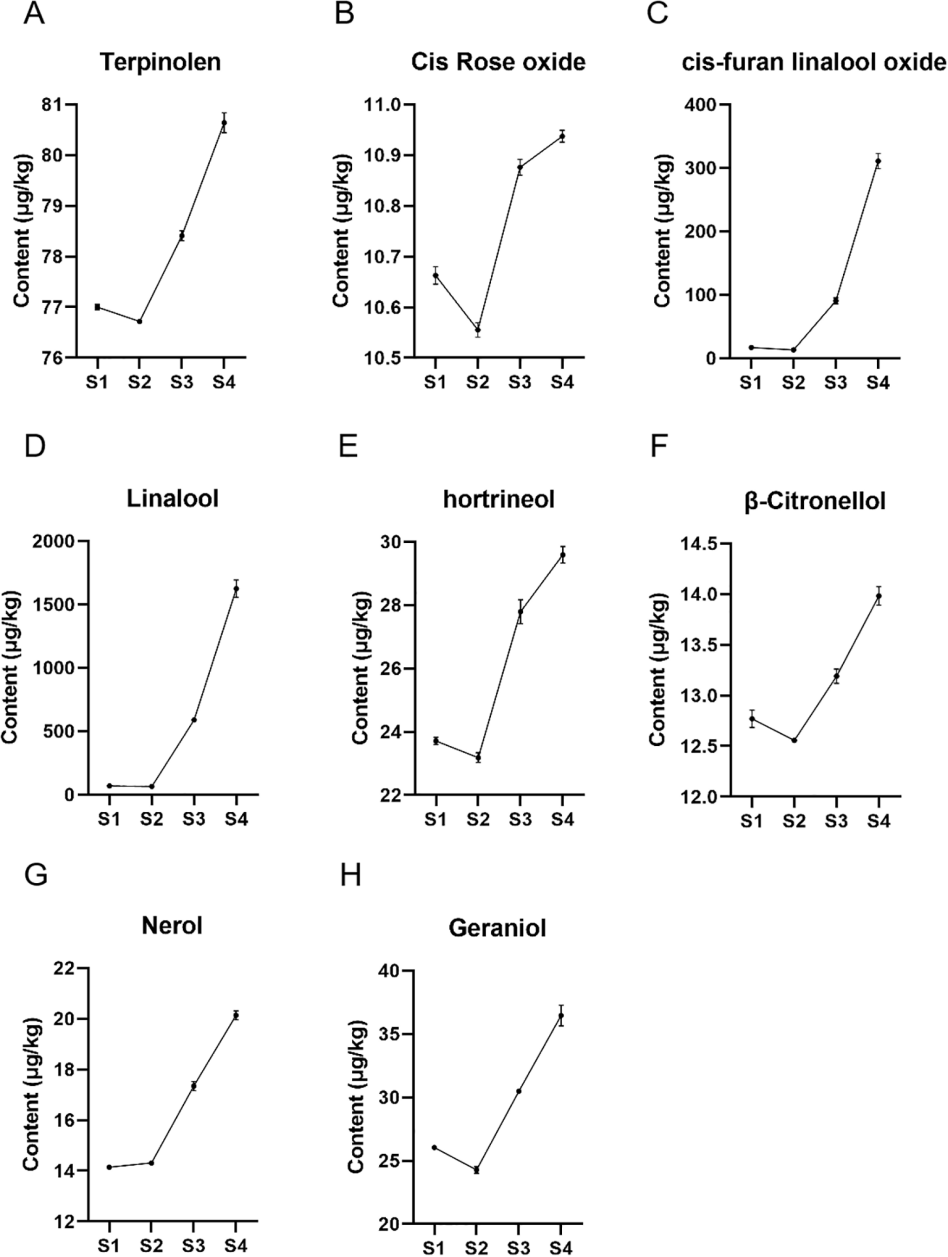
Monoterpene contents of ‘Ruidu Xiangyu’ grape berries in different periods. **(A–H)** 8 monoterpenes (terpinolene, cis-Rose oxide, cis-furan linalool oxide, linalool, hotrienol, β-Citronellol, nerol and geraniol) in developmental stages (S1–S4) of grape fruit. The horizontal axis represents developmental stages (S1–S4), and the vertical axis represents monoterpene concentrations (μg/kg). Data are presented as mean ± SD (n=3 biological replicates).

Since monoterpene metabolites are mainly accumulated in grape berries, we investigated the accumulation of monoterpenes at four stages of grape berry development. The study identified 8 key monoterpene metabolites contained in all 4 developmental stages of ‘Ruidu Xiangyu’ grape berries, including terpinolene, cis-Rose oxide, cis-furan linalool oxide, linalool, hotrienol, β-Citronellol, nerol and geraniol. The results in **Figure 9** show that the contents of the eight monoterpenes showed an overall increasing trend during the S1-S4 period, especially during the S3-S4 period when the rate of increase was more drastic. Specifically, except for nerolidol, the contents of the other seven monoterpenes showed a decreasing trend in the S1-S2 period.

### 3.9 Correlation analysis

**Figure 10:**
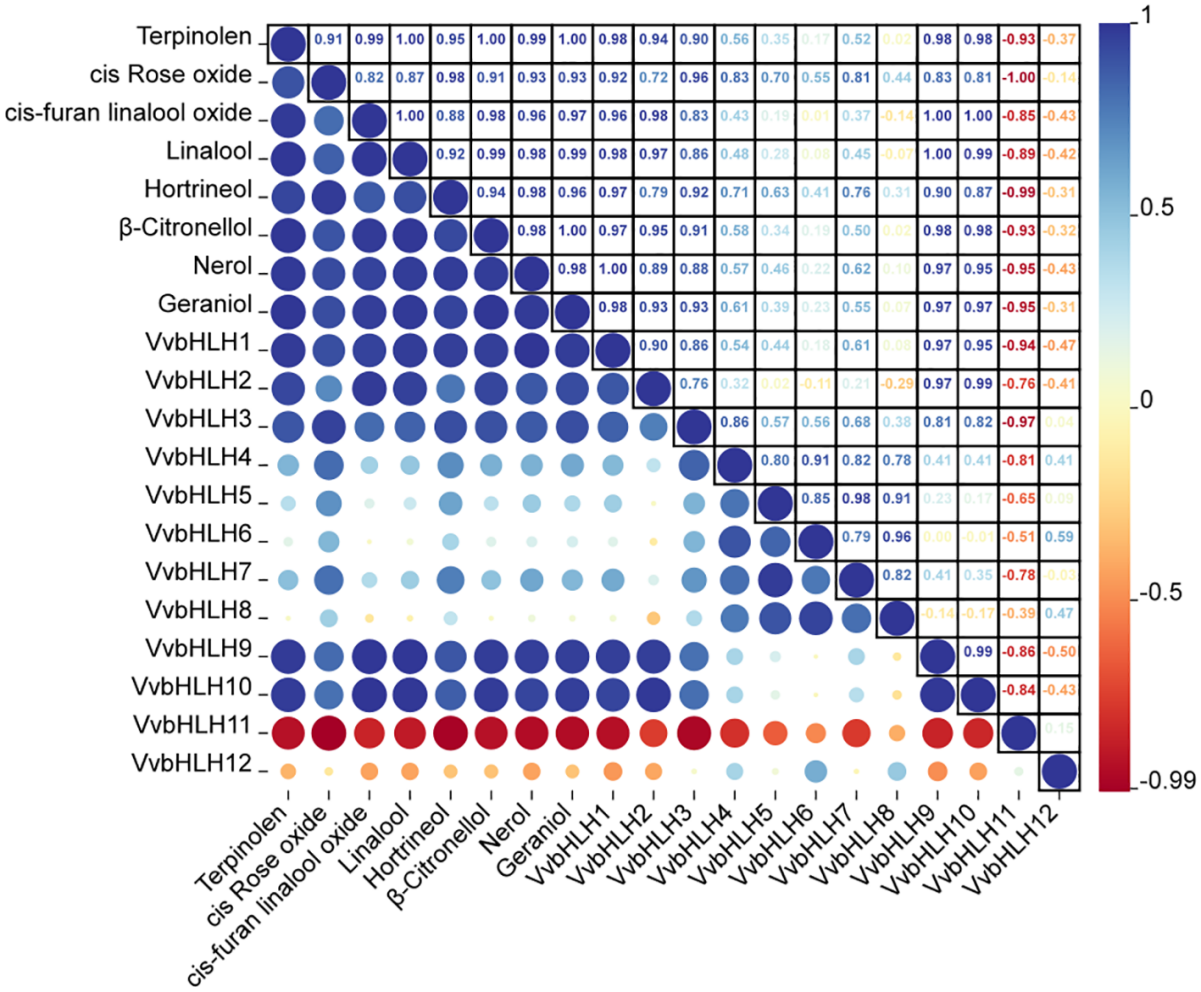
Correlation analysis of monoterpene content and gene expression in fruit of grapes at different periods of time.

Since the monoterpene content in ‘Ruidu Xiangyu’ grape berries increased sharply in the S3 period (color change period), Pearson correlation analysis was conducted to assess the relationship between monoterpene content and the expression levels of the 12 *VvbHLH* genes across the S1–S4 developmental stages. It can be seen that there is also a correlation between the gene expression levels of the bHLH family members in grapes (**Figure 10**). It was found that *VvbHLH1*, *VvbHLH2*, *VvbHLH3*, *VvbHLH9*, and *VvbHLH10* all displayed a pronounced positive linkage with monoterpene concentration in grape berries, while *VvbHLH11* displayed a pronounced negative linkage with monoterpene content in grape berries (**Figure 10**; **Supplementary Table 5**). Specifically, *VvbHLH1* displayed a pronounced positive linkage (P<0.05) with the increase in terpinolene, cis-furan linalool oxide, linalool, hotrienol, β-Citronellol and geraniol content in grapevine fruit, whereas it displayed a pronounced positive linkage (P<0.01) with the increase in nerol content, and *VvbHLH2* displayed a pronounced positive linkage (P<0.01) with the increase in cis-furan linalool oxide and linalool concentration in grapevine fruit. *VvbHLH3* displayed a pronounced positive linkage (P<0.05) with the growth of cis-Rose oxide content. *VvbHLH9* and *VvbHLH10* displayed a pronounced positive linkage (P<0.05) with terpinolene, β-Citronellol, nerol and geraniol content, and strongly positively correlated (P<0.01) with the growth of cis-furan linalool oxide and linalool content. *VvbHLH11* displayed a pronounced negative linkage (P<0.05) for hotrienol content and for cis-Rose oxide content (P<0.01).

### 3.10 Subcellular localization of *VvbHLH9*

**Figure 11:**
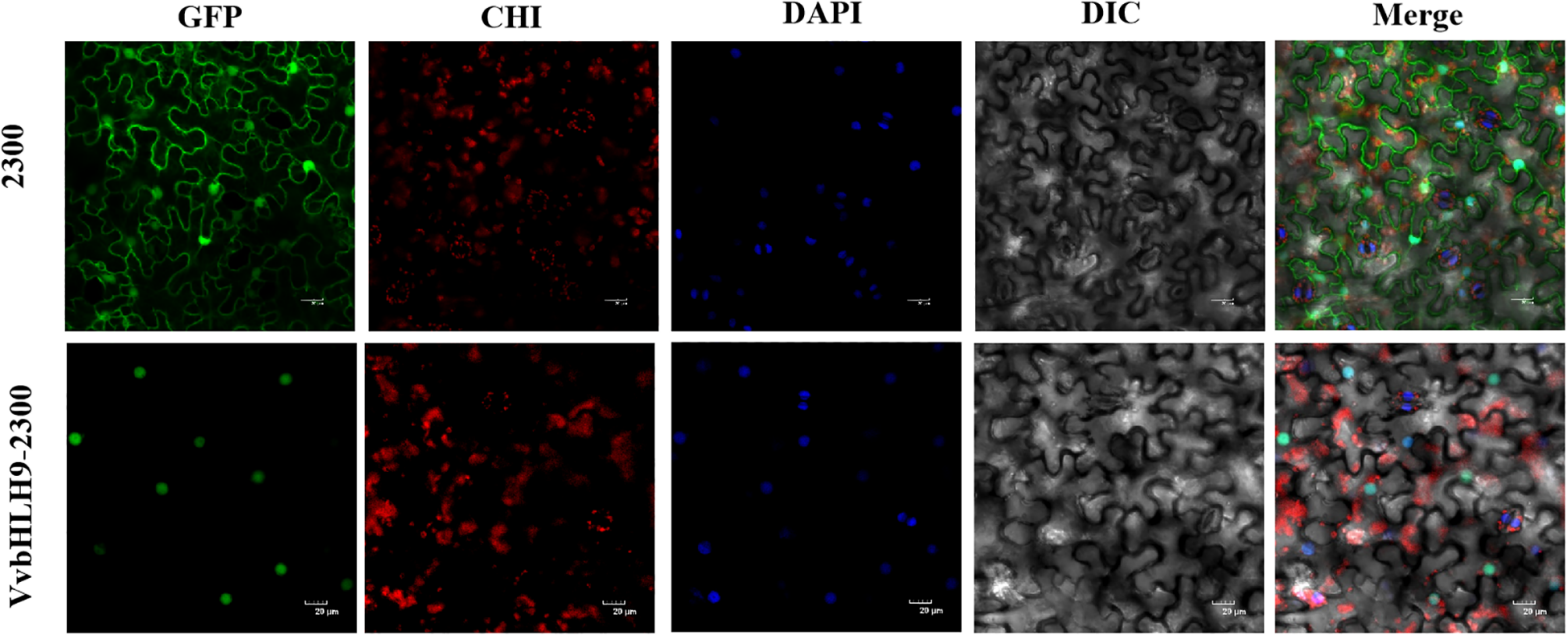
Results of subcellular localization of VvbHLH9 protein in *N. benthamiana.*.

To validate the predicted results of position of VvbHLH9 protein, we successfully cloned the full-length CDSs of *VvbHLH9* gene and ligated them into PCAMBIA2300-GFP vector and transformed them into GV3101 receptor cells. Leaves of *N. benthamiana* were infested using the successfully tested Agrobacterium liquid, and the results were observed. Through subcellular localization, it was found that the green fluorescent protein without insertion of the *VvbHLH9* gene was expressed in various organelles of *N. benthamiana*, but the green fluorescent protein fused with the VvbHLH9 protein was expressed only in the nucleus, which proved that the *VvbHLH9* gene was expressed and functional in the nucleus (**Figure 11**).

### 3.11 Overexpression of *VvbHLH9* promotes the synthesis of grape monoterpene metabolites

**Figure 12:**
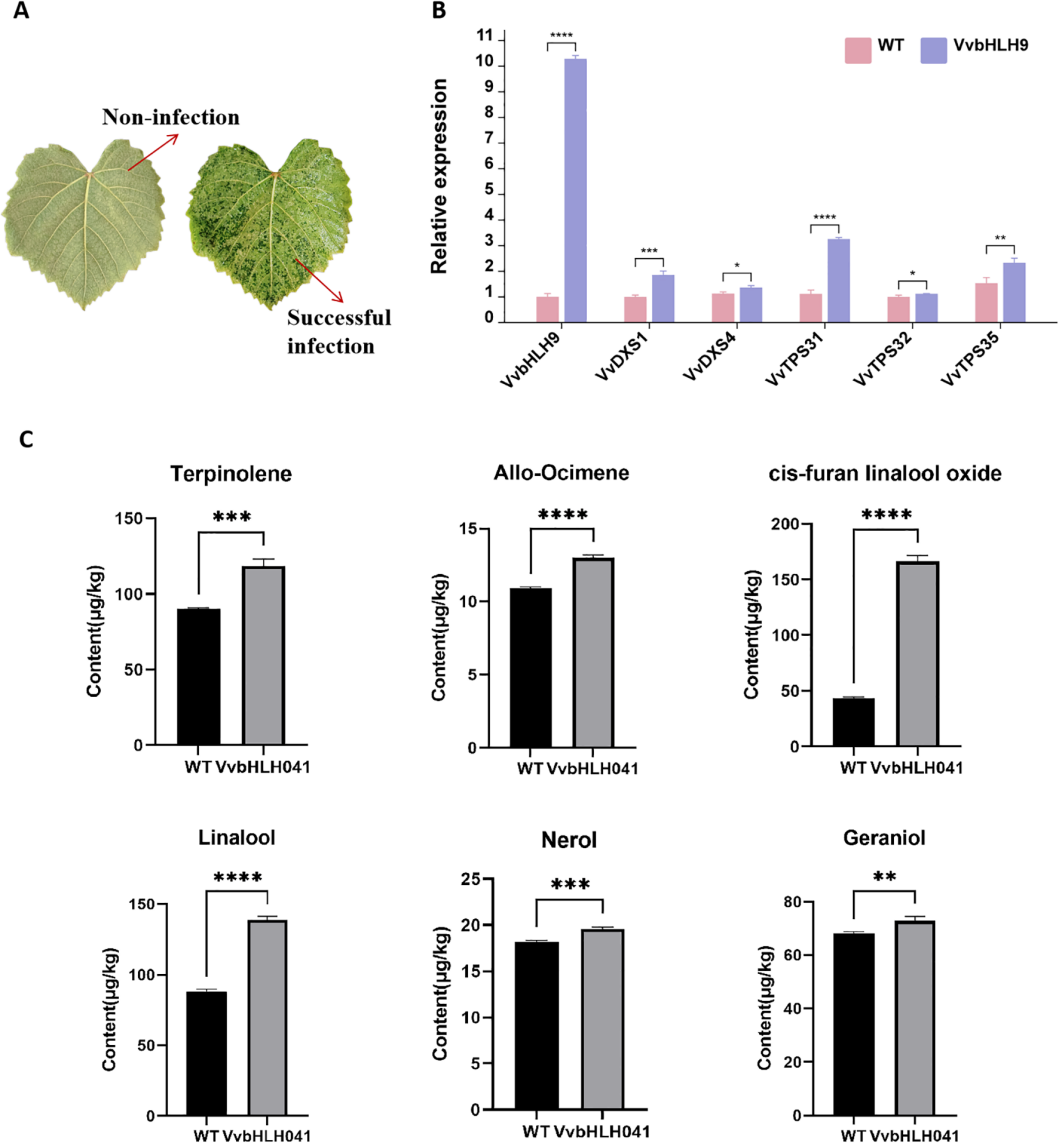
Functional analysis of *VvbHLH9* in ‘Alden’ grape leaves. **(A)** Transient overexpression in grape leaves. Images show uninfected and infected leaves. **(B)** qRT-PCR results of monoterpene synthesis related genes in grape leaves before and after *VvbHLH9* overexpression. Asterisk * represents significant level of p<0.1, asterisk ** represents significant level of p<0.01, asterisk *** represents significant level of p<0.001, and asterisk **** represents significant level of p<0.0001. **(C)** Concentration of monoterpenes (μg/kg) in ‘Alden’ grape leaves. WT represents the wild-type control; VvbHLH9 represents overexpressed VvbHLH9 group from infected leaves.

To further explore the contribution of *VvbHLH9* in grape monoterpene production, the study was carried out to explore the overexpression of *VvbHLH9* gene in ‘Alden’ grape leaves. It could be seen that the color of the infested leaves was significantly darker compared to the color of the non-infested leaves (**Figure 12A**). RT-qPCR data established that the gene markedly elevated the expression of critical enzyme-encoding genes involved in monoterpene formation, *VvDXS1*, *VvDXS4*, *VvTPS31*, *VvTPS32* and *VvTPS35* (**Figure 12B**). Quantification of monoterpene metabolites in grape leaves showed that overexpression of *VvbHLH9* promoted an increase in the content of several monoterpenes including terpinolene, allo-ocimene, cis-furan linalool oxide, linalool, nerol and geraniol, with the increase in cis-furan linalool oxide and linalool being particularly significant (**Figure 12C**). This observation is in line with prior findings that *VvbHLH9* displayed a pronounced positive linkage with the concentration of terpinolene, β-Citronellol, nerol, geraniol, cis-furan linalool oxide and linalool suggesting that *VvbHLH9* positively regulates the synthesis of monoterpenes in grapes.

## 4 Discussion

As widespread transcriptional regulators in plants, the bHLH is strongly conserved in the regulation of metabolism (Wei and Chen, 2018). Carretero-Paulet et al. (2010) conducted a comprehensive phylogenetic analysis of bHLHs from *Arabidopsis*, poplar, rice, moss, and five algae species, classified the 638 identified *bHLHs* into 32 subfamilies, and defined the conserved regions of plant *bHLHs*, this opens up a feasible research pathway for identifying new bHLHs. In this research, the grape bHLHs was identified and analyzed based on the *Arabidopsis* bHLHs, and 12 *VvbHLHs* were identified. Chromosomal localization revealed that the chromosomal distribution of grape bHLH transcription factors was characterized by non-uniformity, which was also present in *Arabidopsis* and apple (Li et al., 2006; Mao et al., 2017). Further covariance analyses showed that VvbHLH family amplification was mainly driven by segmental replication (VvbHLH3/10, VvbHLH4/12), while tandem replication events were not seen, which provided clues for resolving its evolutionary mechanism. Notably, the *VvbHLHs* are mostly located in the nucleus, and their secondary structures are dominated by α-helix and random coil, which are highly similar to the structures of bHLHs from *Arabidopsis* and Ginkgo biloba, suggesting a conserved functional mechanism (Zhou et al., 2020; Liang et al., 2022). Phylogenetic analyses confirms that members with identical intron numbers and conserved motifs tend to cluster preferentially, this result aligns with earlier studies (Ke et al., 2020a). Moreover, grape bHLHs are more closely related to dicotyledonous plants (e.g., apple, pear), further supporting the species-specific association of bHLH family function with evolutionary relationships.

Promoter is a DNA sequence fragment recognized, bound, and transcribed by RNA polymerase, analyzing the cis acting elements of its promoter can help infer the potential function of genes (Hernandez-Garcia and Finer, 2014). Previous studies have shown that the bHLHs are essential for plant adaptation to abiotic stresses (Wu et al., 2022). In wheat, overexpression of the *TabHLH1* can alter stomatal movement, leaf water loss rate, and other growth traits under drought stress in plants, and regulate the abscisic acid pathway to improve drought adaptation (Yang et al., 2016). Our investigation revealed that all 12 *bHLHs* contained hypoxia-specific inducible elements, indicating their potential involvement in stress response mechanisms. In addition, six *VvbHLH* genes (*VvbHLH2*, *VvbHLH4*, *VvbHLH6*, *VvbHLH7*, *VvbHLH8*, and *VvbHLH9*) were able to participate in the regulation of zeinolysin metabolism in plants, which suggests that *VvbHLHs* are involved in metabolic regulatory processes in plants. Previous studies have shown that *MdbHLH3* in apple directly binds to the promoter of *MdcyMDH* and activates its transcriptional expression, which promotes malic acid accumulation in fruits (Yu et al., 2021). Increased expression of the bHLH *AtMYC2* in *Arabidopsis* substantially increased the number of glandular hairs, which in turn promoted the synthesis of terpenes in glandular hairs (Zhai et al., 2013). The results verify that bHLH family members serve as key mediators of metabolic network regulation in planta.

Plant bHLH transcription factors often collaborate with other regulatory proteins to control the synthesis of diverse specialized metabolites, including terpenoids, phenylpropanoids, and flavonoids, and are critical for mediating plant-environment interactions (Feller et al., 2011). Terpenoids represent essential metabolic products that are ubiquitously present in plants. Existing research has demonstrated that bHLHs are significant regulatory factors in plant terpenoid biosynthesis (Yi et al., 2022). As a economically significant fruit species, grape quality attributes are strongly associated with terpenoid biosynthesis pathways (Zhang et al., 2024b). For investigating the impact of *VvbHLHs* on monoterpenoid synthesis patterns in grapevine, the study choose the juicy ‘Ruidu Xiangyu’ berries as the test material, and correlation analyses of gene expression and monoterpene content during the developmental period of the berries were carried out. The results showed that *VvbHLH11* and *VvbHLH12* were mainly expressed at the early stage of fruit development, a result consistent with the tendency of *CmbHLH32* in melon to be expressed at the early stage of fruit development (Tan et al., 2021). In addition, in this study, five *VvbHLHs* (*VvbHLH1*, *VvbHLH2*, *VvbHLH3*, *VvbHLH9*, and *VvbHLH10*) had similar expression patterns, all of which demonstrating a marked upregulation in their expression during fruit ripening, these results are consistent with the functions of some *bHLHs* in grape, apple, and peach, indicating their potential importance in fruit ripening. It is hypothesized that such *bHLHs* may be the key genes driving the marked increase in monoterpene levels in fruits (Yang et al., 2017; Wang et al., 2018; Zhang et al., 2018a). Notably, *VvbHLH11* expression showed a significant negative correlation with monoterpene accumulation, suggesting its potential role as a repressor in monoterpene biosynthesis. This inhibition might occur through competitive interference with activator-type bHLHs or direct downregulation of *VvTPS* genes. A comparable regulatory mechanism has been observed in *Salvia miltiorrhiza*, where SmbZIP1 suppresses tanshinone biosynthesis by inhibiting *SmGGPPS* expression (Deng et al., 2020). Further validation through *VvbHLH11* knockout or ChIP assays would clarify its repressive mechanism in grape monoterpene synthesis.

Significantly, our investigation revealed a gene situated on chromosome 2, designated as *VvbHLH3* (Vitvi02g00231), which encodes the transcription factor MYC1. Extensive evidence has revealed that MYC1 serves as an essential regulator in the metabolic pathways of flavonoids and terpenoids (Quattrocchio et al., 2006; Hichri et al., 2010; Muhammad et al., 2023; Li et al., 2024; Yu et al., 2024). In plants such as maize, petunia and *Arabidopsis*, MYC1 can promote flavonoid production by binding with MYB (Quattrocchio et al., 2006; Muhammad et al., 2023). In grape, *VvMYC1* by itself does not activate the promoters of the *VvCHI* and *VvUFGT* of the flavonoid biosynthesis pathway, but its synergistic interaction with MYB significantly enhances promoter activity (Hichri et al., 2010). The current study identified *VvMYC1* as a novel regulator of grape monoterpene metabolism, suggesting that a similar synergistic regulatory mechanism may also operate in this pathway. In addition, Yu et al. (2024) found that tomato *SlMYC1* mediated trichome development and terpene metabolite synthesis by directly binding to the promoter of *SlTOR* and activating its expression, Our spatiotemporal expression studies demonstrated that the gene was significantly induced during the stage of highest monoterpene synthesis in grapes (from veraison to ripening), and displayed a pronounced positive linkage with the concentration of cis-Rose oxide monoterpenes. The above functional diversity indicates that bHLHs likely participate in modulating diverse secondary metabolic processes via a complex interaction network. However, the mechanism regarding the regulation of grape monoterpene production by MYC-like bHLH transcription factors is not yet fully understood and needs to be further clarified by means of molecular biology studies such as yeast single-hybrid and dual-luciferase reporter experiments.

Unlike the conserved structural domains of the remaining 11 VvbHLH transcription factors in this study, VvbHLH9 (annotated as bHLH041 in the NCBI RefSeq database, accession XP_002279486.2) does not contain the bHLH-MYC_N structural domain but retains the intact bHLH_AtbHLH_like structural domain, which drew our attention. Previous studies on terpene biosynthesis-associated *bHLHs* in other plants (e.g., *Arabidopsis* and tomato) have been relatively scarce, focusing mainly on MYC2 (Kazan and Manners, 2013; Spyropoulou et al., 2014). Since then, researchers have identified MYC2-independent bHLHs (BIS1 and BIS2), in the medicinal plant periwinkle, which both activate the monoterpene formation of the MIA (Liu et al., 2021). This investigation uncovered high expression of *VvbHLH9* during grape ripening and its strong correlation with the accumulation of key monoterpenes (e.g., linalool, terpinolene) suggests that it orchestrates monoterpene synthesis fluxes through developmental stage-specific transcription. To further validate the function of *VvbHLH9* in monoterpene synthesis, subcellular localization of the gene was investigated and transgenic lines overexpressing the gene were established in ‘Alden’ grape leaves. The results confirmed that *VvbHLH9* exerted its transcriptional regulatory function mainly in the nucleus and significantly activated the transcription of genes such as *VvDXS1* and *VvTPS31*, and positively regulated the biosynthesis of a variety of monoterpene metabolites including linalool. This suggests that *VvbHLH9* may exert regulatory effects on monoterpene metabolites by activating upstream synthetic modules. However, given that this gene lacks a typical MYC structural domain, whether its regulation is achieved through the formation of heterodimers or modification of chromatin state still needs to be further resolved by Co-IP combined mass spectrometry analysis.

## 5 Conclusion

This research carried out a systematic genomic characterization of the grapevine bHLHs through bioinformatics, with the goal of screening for *VvbHLH* genes linked to monoterpene production. The study identified 12 key *bHLHs* genes in the grapevine genome. The results of covariance analysis indicated intraspecific covariance between two pairs of *VvbHLHs* and interspecific covariance between eight pairs of *VvbHLHs* and *Arabidopsis bHLHs*. Using phylogenetic reconstruction, 12 VvbHLHs were divided into four main subfamilies. RT-qPCR results demonstrated that the *VvbHLHs* were expressed in different developmental periods in grape and showed spatio-temporal specificity. By correlating grape *VvbHLHs* with the results of monoterpene content measurements, five *VvbHLHs* (*VvbHLH1*, *VvbHLH2*, *VvbHLH3*, *VvbHLH9*, and *VvbHLH10*) were found to be closely related to a dramatic growth in monoterpene concentration. Among them, *VvbHLH9* (*VvbHLH9*) was shown to be associated with the accumulation of monoterpene metabolites such as terpinolene, allo-ocimene, nerol, geraniol, cis-furan linalool oxide and linalool in grapevines. The investigation sheds light on the whole-genome identification of grape bHLHs and their evolutionary history, and has important implications for quality improvement and molecular breeding in grape.

## Data Availability

Existing datasets are available in a publicly accessible repository:Publicly available datasets were analyzed in this study. The original proteome file of Vitis vinifera can be found in the Ensembl Plants repository here: [https://ftp.ebi.ac.uk/ensemblgenomes/pub/release-59/plants/fasta/vitis_vinifera/pep/]. The protein sequences of Arabidopsis thaliana bHLH transcription factors were obtained from the PlantTFDB database: [https://planttfdb.gao-lab.org/tf.php?sp=Ath&did=AT1G01260.1].
